# Sensation Seeking and Adaptation in Parabonauts

**DOI:** 10.3389/fpsyg.2018.00296

**Published:** 2018-03-09

**Authors:** Aurélie Collado, Jean-Philippe Hainaut, Vincent Monfort, Benoît Bolmont

**Affiliations:** ^1^Université de Lorraine, 2LPN-CEMA Group (Cognition-EMotion-Action), EA 7489, Dept Sport Sciences, Metz, France; ^2^Université des Antilles, ACTES (UPRES EA 3596), UFR STAPS, Pointe-à-Pitre, France

**Keywords:** adaptation, parabolic flights, microgravity, sensation seeking, coping strategies, motion sickness susceptibility, parabonauts’ characteristics, Zero-G fliers

## Abstract

Evidence from extreme environments suggests that there are relationships between difficulties of adaptation and psychological factors such as personality. In the framework of microgravity research on humans, the aim of this exploratory study was to investigate inter-individual differences of parabonauts on the basis of quality of adaptation to the physical demands of parabolic flights. The personality characteristics of two groups of parabonauts with a different quality of adaptation (an Adaptive group, *N* = 7, and a Maladaptive group, *N* = 15) were assessed using the Sensation Seeking Scale, Brief COPE, and MSSQ-Short. Compared to the Maladaptive group, the individuals of the Adaptive group scored higher on Boredom Susceptibility (i.e., a subscale of the Sensation Seeking Scale), lower on scales of susceptibility to motion sickness (MSSQ-Short) and tended to score lower on Instrumental Support Seeking (i.e., a subscale of the Brief COPE). These results suggest that individuals of the Adaptive group are more intolerant to monotony, present an aversion to repetitive and routine activities, are less susceptible to motion sickness and less dependent on problem-focused strategies. These characteristics may have contributed to developing a certain degree of flexibility in these subjects when faced with the parabolic flight situation and thus, may have favored them. The identification of differences of personality characteristics between individuals who have expressed difficulties of adaptation from those who have adapted successfully could help to prevent the risk of maladaptation and improve the well-being of (future) commercial or occupational aerospace passengers. More generally, these results could be extended to extreme environments and professional and/or sports domains likely to involve risk taking and unusual situations.

## Introduction

Described as mimicking spaceflight-associated conditions (e.g., Strewe et al., 2012), parabolic flights constitute the best ecological model on earth to investigate the effects of microgravity and/or different gravity transitions and, thus, to study human adaptation to these physical demands presented by the space environment. In fact, microgravity and gravitational changes that involve unique physical demands lead to perceptual mismatches between various information from the vestibular system on the one hand (i.e., canal-otolith conflict) and between information from the visual system and from the vestibular system on the other hand (i.e., visuo-vestibular conflict) (Reason, 1978; Benson, 2002; Baker et al., 2008; Schmäl, 2013). These sensory conflicts can induce maladaptation (i.e., motion sickness symptoms) and seem to affect people differently (Reason and Brand, 1975; Davis et al., 1988; Reschke, 1990; Lackner and DiZio, 2006; Golding et al., 2017). In fact, studies carried out in a microgravity environment have reported differences not only in the frequency of appearance of maladaptation but also in their severity (e.g., Davis et al., 1988; Benson, 2002; Golding et al., 2017). Consequently, these studies suggest the existence of individual differences faced with the physical demands of the unusual environment (i.e., microgravity and gravitational changes). Interestingly, it should be noted that ground-based studies in the context of a broader field of research have suggested an influence of psychological factors such as dispositional characteristics in adaptation to the physical demands of the environment (e.g., Collins and Lentz, 1977; Bick, 1983; Fox and Arnon, 1988; Gordon et al., 1994; Paillard et al., 2013). Among dispositional characteristics, personality has been studied in relation to the difficulties of adapting to a physical environment on earth (i.e., susceptibility to air sickness, seasickness, etc.). On the basis of several studies, evidence suggests a relationship between the characteristics of personality and difficulties of adaptation to a physical environment (Collins and Lentz, 1977; Bick, 1983; Gordon et al., 1994; Paillard et al., 2013). Moreover, studies on other extreme environments highlight the fact that personality could influence the extent to which an individual adapts effectively. Although each extreme environment contains unique physical (and social) demands, some characteristics such as emotional instability, high neuroticism, sensation seeking or anxiety seem to be unfavorable overall to adapting (Gunderson, 1974; Steel et al., 1997; Abraini et al., 1998; Palinkas et al., 2000; Palinkas and Suedfeld, 2008; Lafère et al., 2017). Consequently, personality plays a crucial role in the adaptation process. It can therefore compromise this adaptation process (e.g., Sandal et al., 1996; Palinkas et al., 2000; Bolmont et al., 2001; Bolmont and Collado, 2014).

In parabolic flight situations, recent studies have investigated the psychological factors affecting people participating in parabolic flights (i.e., parabonauts) in order to try to identify possible predictors of maladaptation on one hand (e.g., Choukèr et al., 2010; Strewe et al., 2012; Van Ombergen et al., 2016; Collado et al., 2017; Golding et al., 2017) and to better describe this specific population on the other hand (e.g., Collado et al., 2014; Montag et al., 2016). Most studies carried out on possible predictors of maladaptation signs have mainly focused on the situational characteristics of the voluntary participants (Choukèr et al., 2010; Strewe et al., 2012; Van Ombergen et al., 2016; Collado et al., 2017; Golding et al., 2017). It should be noted that few studies have investigated the dispositional characteristics of people participating in parabolic flight and have highlighted a specific personality profile characterizing parabonauts (Collado et al., 2014; Montag et al., 2016). Parabonauts appear to be stimulation seekers who are conscientious, emotionally stable, less anxious and who tolerate stress better than the general population (Collado et al., 2014) or than a control group (Montag et al., 2016). In their study, Collado et al. (2014) revealed that people attracted by parabolic flights scored higher on Extraversion and differed in four out of six NEO-PI-R facets of this domain (e.g., Activity, Excitement-Seeking, Positive Emotions). These distinctive facets suggest that voluntary participants have a more rapid pace of living, and need to be more stimulated by the environment than the general population. Given that parabonauts need to be permanently stimulated by their environment, and as suggested previously (Collado et al., 2014), it would be interesting to focus on sensation seeking in parabonauts in order to examine whether this dispositional characteristic can provide information on quality of adaptation to the physical demands of parabolic flights. Interestingly, a study on major affective disorders has shown that a sensation seeking pattern could predict hyperthymic temperament (Engel-Yeger et al., 2016), a personal disposition with “positive” traits such as being optimistic, fun-loving, confident, outgoing, jocular, on the go but also being a risk-taker (Akiskal et al., 2005). It should be noted that sensation seeking has been investigated in a recent parabolic flight study (Montag et al., 2016). However, the authors only assessed this dispositional characteristic in a control group without the possibility of considering parabonauts.

Considering the unique physical demands of parabolic flights that are likely to hinder adaptation and the involvement of the personality in the adaptation process on the one hand (Sandal et al., 1996; Palinkas et al., 2000; Bolmont and Collado, 2014), and given the involvement of personality domains in dispositional coping on the other (e.g., Costa et al., 1996; Watson and Hubbard, 1996; Ferguson, 2001), the main objective of this exploratory study is to identify differences in dispositional characteristics such as sensation seeking or trait coping strategies on the basis of the quality of adaptation (successfully adapted or not) to the physical demands of parabolic flights. In the present study, our hypothesis was that parabonauts who have expressed difficulties adapting could present differences in trait-coping strategies or subscales of sensation seeking that are likely to hinder their adaptation compared to parabonauts who have adapted successfully.

## Materials and Methods

### Participants

The data presented in this study were drawn from a larger *ETAP-0g Project* study, which investigated behavioral, psychological, and physiological parameters during parabolic flights. Data was collected over 2 parabolic flight campaigns, scheduled between 2010 and 2011. The study was approved in advance by the CNES and the local institutional ethics committee (CPP OUEST II-Angers; approval no. 2007/18). Participants were informed about the experimental procedure and the parabola profile. They were also notified that they were voluntary, anonymous and that their data were protected by the applicable legislation. Each subject was then asked to fill out an informed consent form in accordance with the Declaration of Helsinki, and all participants provided this written consent before participating. The selection criteria for the *ETAP-0g Project* study were as follows: subjects had to be healthy men, with at least a second-year university level education, have no previous experience in parabolic flight, no history of severe motion sickness, no history of psychiatric, neurological or vestibular disorders, and comply with the medical requirements for parabolic flights. During the parabolic flight, all participants were only “subjects of the experiment” to the exclusion of any other role, and were assigned to perform the same tasks with relatively simple reaction times under the same conditions, i.e., to press a response-button as quickly as possible as soon as they perceived a stimulus (results presented below). Caffeine and alcohol were strictly prohibited 24 h before the beginning of the flight. No anti-emetics were used before or during the flight.

A total of 24 participants were involved in this study (mean age: 24.71 ± 4.88 year). In order to recruit a large group for this exploratory study, the sex variable was excluded. Because women respond to stress differently from men (e.g., intra-individual hormonal variability) and present more limitations for participation in parabolic flights (i.e., risk of pregnancy), only men were recruited to participate in this study during parabolic flights.

### Assessment

Personality characteristics and susceptibility to motion sickness were assessed on the basis of forms filled out after the parabolic flights during the laboratory session (second phase) of the ETAP-0g Project. The objective was to limit response biases (i.e., to eliminate individuals who could respond in an overly desirable manner in order to be selected for this experiment).

#### Sensation Seeking Scale

Sensation seeking was assessed by the Zuckerman’s Sensation Seeking Scale-V (Zuckerman et al., 1978; French version by Carton et al., 1992) which consists of 40 items in which participants have to choose between two statements per item. The Sensation Seeking Scale has four item subscales, each of them ranging from 0 to 10: (1) Disinhibition (i.e., adoption of socially “uninhibited” and extraverted behaviors, seeking stimulation through various sexual experiences or psychoactive substances), (2) Thrill and Adventure Seeking (i.e., a set of sports and activities that include a risk-taking dimension), (3) Experience Seeking (i.e., seeking an unconventional lifestyle and new sensory or intellectual experiences), and (4) Boredom Susceptibility (i.e., intolerance to monotony manifested by an aversion to repetitive and routine activities). In the present sample, the Sensation Seeking Scale-V was characterized by a Cronbach’s alpha of 0.81. The subscales ranged from 0.51 to 0.71.

#### Brief COPE

Trait coping was assessed by the Brief COPE (Carver, 1997; French version by Muller and Spitz, 2003) which consists in a self-evaluation questionnaire about the usual way individuals deal with the stressors of everyday life. The Brief COPE consists of 28 items divided into 14 scales allowing assessment of 14 distinct dimensions of coping: (1) active coping, (2) planning, (3) using instrumental support, (4) using emotional support, (5) venting, (6) behavioral disengagement, (7) self-distraction, (8) self-blame, (9) positive reframing, (10) humor, (11) denial, (12) acceptance, (13) religion, and (14) substance use. Each items scored on a four-point scale and scores for each dimension have a range from 2 to 8. The Brief COPE was characterized by a Cronbach’s alpha of 0.72. Internal consistencies of the questionnaire were satisfactory ranging from 0.58 to 1.00. Self-blame and Acceptance were excluded from analysis, because of low internal consistencies (i.e., <0.5).

#### MSSQ-Short

Susceptibility to motion sickness was assessed by the MSSQ-Short questionnaire (i.e., Motion Sickness Susceptibility Questionnaire Short-form, Golding, 1998; French version by Paillard et al., 2013). This short self-assessment questionnaire consists of two parts evaluating the general experience of motion sickness symptoms in childhood (before the age of 12 years, Child section A, i.e., MSA score) and in the last 10 years (section Adult section B, i.e., MSB score). The subject must indicate how often he has felt sick or nauseated in different situations (e.g., cars, trains, ships, swings, and roundabouts in playgrounds, fairground rides...) by varying their intensity on the following scale: “Not Applicable – Never Travelled,” “Never Felt Sick,” “Rarely Felt Sick,” “Sometimes Felt Sick,” and “Frequently Felt Sick.” MSA and MSB raw scores range from 0 to 27 and add up to give a total MSS score that ranges from 0 to 54. Cronbach’s alpha was 0.83.

#### Maladaptation/Adaptation

Maladaptation during parabolic flights was assessed after the flight by questionnaires listing potential symptoms of motion sickness (sweating, drowsiness, headache, stomach discomfort, nausea, vomiting), their frequency and severity. Self-reported symptoms in the form of observations by subjects were collected and compared to the experimenters’ observations in order to check data consistency. Because this study was exploratory, frequency, and severity were only noted in order to remove any doubt about identification of the occurrence of adaptation difficulties. Data from two participants were removed from the analyses due to their inability to perform required reaction time tasks during parabolic flights. From this cohort (*n* = 22), two groups were constituted. The subjects who presented signs of maladaptation with at least one symptom of motion sickness constituted the Maladaptive group (25.33 ± 5.63 year; *n* = 15). The others formed the Adaptive group (23.00 ± 3.46 year; *n* = 7).

### Procedure

Experiments were performed during parabolic flight campaigns (three flights per campaign) aboard the A300 ZeroG (Bordeaux International Airport, France). These flights are funded by the CNES (*Centre National d’Etudes Spatiales*: French national space research center) and organized by Novespace. They are run under the authority of the *Centre d’Essais en Vol*. The parabolic flights have a standard profile defined by Novespace. They each last about two and half hours (between 9:30 and 12:00) and consist in 30 experimental parabolas preceded by a preliminary test parabola. The parabolas are executed in sets of five with 90 s intervals between parabolas and with 4–8 min intervals between sets of parabolas. A parabolic flight maneuver is characterized by gravitational changes from 1 to 1.8G to 0G to 1.8G to 1G. Each change lasts approximately 20–30 s. Consequently, each parabola lasts approximately 70 s. The complete parabola is followed by 90 s of flight at 1G level.

### Statistical Analyses

Shapiro–Wilk and Levene tests were used to check the normality of distribution and the homogeneity of variance respectively. Because the assumption of normality of distribution and/or homogeneity of variance was contradicted (except for demographic characteristics for which a Student test were applied), non-parametric analyses were conducted in order to determine whether differences existed between the groups. Comparisons between these groups were carried out using a Mann–Whitney *U*-test in order to determine whether differences existed in sensation seeking, coping strategies, and motion sickness susceptibility. Logistic regression was carried out to test the existence of possible predictors for the binary variable “successfully adapted” versus “not successfully adapted.” Because this study was exploratory, and given the sample for all subscales tested that was too small, only the most relevant characteristics for which the inter-group difference was significant were considered. For all statistical analysis, we considered *p*-values less than 0.05 to be statistically significant.

## Results

### Demographic Characteristics

The demographic characteristics of the Adaptive group and the Maladaptive group are presented in **Table 1**. No inter-group differences were found for age (*t* = -0.63; *ns*, η^2^ = 0.05) and Trait-Anxiety (*t* = -1.00; *ns*, η2 = 0.02) assessed by the YA form of the Spielberger State-Trait Anxiety Inventory (i.e., STAI; Spielberger et al., 1983).

**Table 1:** Demographic characteristics by group.

	Adaptive group (*n* = 7)	Maladaptive group (*n* = 15)
	
	Mean ±*SD*	Mean ±*SD*
Age (years)	23.00 ± 3.46	25.33 ± 5.63
Trait-Anxiety	34.71 ± 5.06	36.53 ± 6.84

*Means and standard deviations for the Age and the Trait-Anxiety (STAI) scores of the Adaptive group and the Maladaptive group.*

### Sensation Seeking

**FIGURE 1:**
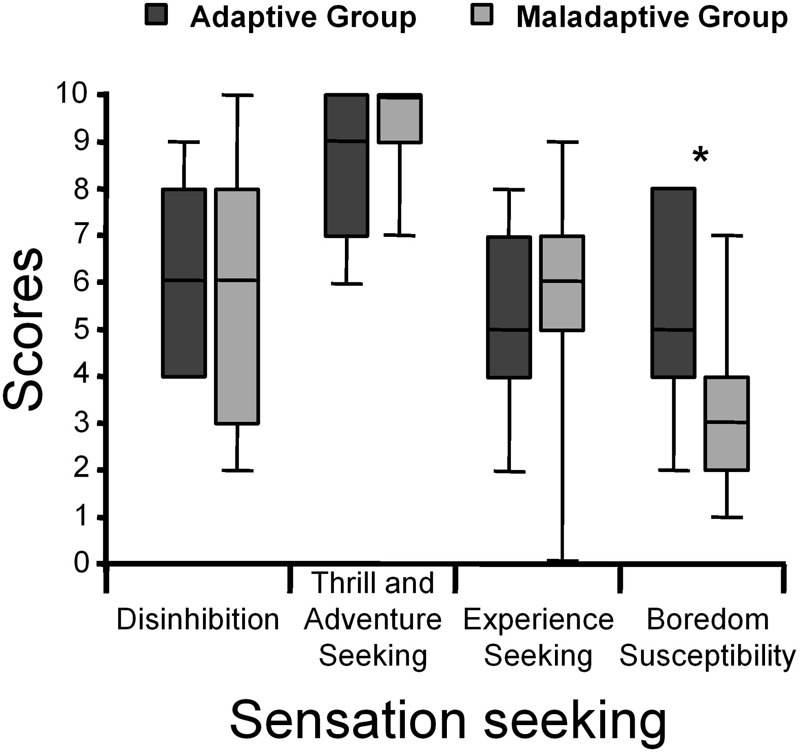
Medians and interquartile ranges of Sensation Seeking subscales in the Adaptive group and the Maladaptive group. Significant differences between the scores of the Adaptive group and the Maladaptive group have been marked as follows: ^∗^*p* < 0.05.

The differences between the Adaptive group and the Maladaptive group on the Sensation Seeking Scale are presented in **Figure 1**. The results showed a significant difference in one out of four subscales. The Adaptive group scored higher than the Maladaptive group on Boredom Susceptibility (*U* = 24.5, *z* = 2.01, *p* < 0.05, η^2^ = 0.21). No significant differences were found for Disinhibition (*U* = 42.5, *z* = 0.71, *ns*, η^2^ = 0.02), Thrill and Adventure Seeking (*U* = 43.5, *z* = -0.69, *ns*, η^2^ = 0.05), and Experience Seeking (*U* = 37.5, *z* = -1.07, *ns*, η^2^ = 0.04).

### Trait Coping

The differences between the Adaptive group and the Maladaptive group in the framework of the dimensions of the Brief COPE are presented in **Table 2**. A trend was observed for coping Using instrumental support. Individuals in the Maladaptive group scored higher than those in the Adaptive group (*U* = 27, *z* = -1.94, *p* = 0.078, η^2^ = 0.17). No significant differences were found for Active coping (*U* = 42.5, *z* = -0.75, *ns*, η^2^ = 0.03), Planning (*U* = 47, *z* = -0.41, *ns*, η^2^ = 0.02), Using emotional support (*U* = 43, *z* = -0.69, *ns*, η^2^ = 0.04), Venting (*U* = 52, *z* = 0.04, *ns*, η^2^ < 0.01), Behavioral disengagement (*U* = 34, *z* = -1.43, *ns*, η^2^ = 0.07), Self-distraction (*U* = 45, *z* = 1.46, *ns*, η^2^ = 0.10), Positive reframing (*U* = 47.5, *z* = -0.37, *ns*, η^2^ = 0.01), Humor (*U* = 46.5, *z* = 0.44, *ns*, η^2^ = 0.02), Denial (*U* = 42, *z* = -1.24, *ns*, η^2^ = 0.06), Religion (*U* = 48, *z* = 0.37, *ns*, η^2^ < 0.01), and Substance use (*U* = 49, *z* = -0.68, *ns*, η^2^ = 0.02).

**Table 2:** Comparison of Brief COPE between Adaptive group and Maladaptive group.

	Adaptive group (*n* = 7)	Maladaptive group (*n* = 15)	*p*-Value
	
	Median (IQR)	Median (IQR)	
Active coping	6.00 (6.00–6.50)	6.00 (6.00–8.00)	*ns*
Planning	6.00 (6.00–7.00)	6.00 (6.00–7.50)	*ns*
Using instrumental support	4.00 (4.00–4.00)	5.00 (4.00–6.00)	*ns*
Using emotional support	6.00 (4.00–7.50)	7.00 (6.00–7.00)	*ns*
Venting	6.00 (5.00–7.50)	6.00 (5.50–7.00)	*ns*
Behavioral disengagement	4.00 (3.50–4.00)	4.00 (4.00–5.00)	*ns*
Self-distraction	2.00 (2.00–2.00)	2.00 (2.00–2.00)	*ns*
Positive reframing	4.00 (3.00–4.50)	4.00 (3.00–5.50)	*ns*
Humor	5.00 (4.50–6.00)	5.00 (4.00–6.00)	*ns*
Denial	2.00 (2.00–2.00)	2.00 (2.00–2.00)	*ns*
Religion	2.00 (2.00–3.50)	2.00 (2.00–3.00)	*ns*
Substance use	2.00 (2.00–2.00)	2.00 (2.00–2.00)	*ns*

*Medians and interquartile ranges (IQR) for the Brief COPE scores of the Adaptive group and the Maladaptive group.*

### Motion Sickness Susceptibility

Differences between the Adaptive group and the Maladaptive group for the MSSQ-Short are presented in **Table 3**. The results showed significant differences in the MSA score (Childhood), the MSB score (Adulthood) and Total MSS score. Compared with the Adaptive group, individuals in the Maladaptive group scored higher on the MSA score (*U* = 21, *z* = -2.26, *p* < 0.05, η^2^ = 0.22), the MSB score (*U* = 16.5, *z* = -2.67, *p* < 0.01, η^2^ = 0.16) and the Total MSS score (*U* = 17, *z* = -2.52, *p* < 0.05, η^2^ = 0.22).

**Table 3:** Comparison of Motion Sickness Susceptibility between the Adaptive group and the Maladaptive group.

	Adaptive group (*n = 7*)	Maladaptive group (*n = 15*)
	
	Median (IQR)	Median (IQR)
MSA score (Child section)	0.00 (0.00–2.25)	5.63 (1.75–7.71)^∗^
MSB score (Adult section)	0.00 (0.00–0.00)	1.29 (0.50–2.50)^∗∗^
Total MSS score	1.00 (0.00–2.25)	6.75 (2.50–8.92)^∗^

*Medians and interquartile ranges (IQR) for the Motion Sickness Susceptibility Questionnaire Short-form (MSSQ-Short) scores of the Adaptive group and the Maladaptive group. ^∗^Significantly different from the Adaptive group (*p* < 0.05). ^∗∗^Significantly different from the Adaptive group (*p* < 0.01).*

### Predictors of Maladaptation Signs

The total MSS score and the Boredom Susceptibility score were included in the logistic regression model. The logistic regression model (χ^2^ = 13.26, df = 2, *p* < 0.001; Nagelkerke *R*^2^ = 0.63) gave 93.33% correct classification for “not successfully adapted” and showed that “not successfully adapted” was predicted by the Total MSS score (*p* < 0.01) and the Boredom Susceptibility (*p* < 0.05).

## Discussion

The objective of this exploratory study was to identify differences in dispositional characteristics such as sensation seeking or trait coping strategies according to the quality of adaptation (successfully adapted or not) to the physical demands of parabolic flights. Compared to the individuals in the Maladaptive group, those in the Adaptive group scored higher on Boredom Susceptibility (i.e., a subscale of the Sensation Seeking Scale) and lower on scales of susceptibility to motion sickness. A low level of Boredom Susceptibility and a high Total MSS score were found to predict Maladaptive group membership. No significant differences were found in the subscale of the Brief COPE, except for a trend in Instrumental Support Seeking (i.e., *p* = 0.078)—a problem-focused strategy that corresponds to seeking information, assistance and/or advice (Muller and Spitz, 2003) with a higher score for the Maladaptive group compared to the Adaptive group.

With respect to the scales of susceptibility to motion sickness (i.e., MSSQ), the Adaptive group showed a lower score in the raw score, the childhood and the adulthood section compared to the Maladaptive group. These results seem to be consistent with the distinction criteria applied in this study in order to separate individuals with adaptation difficulties from those who have successfully adapted to parabolic flight conditions. This distinction criterion was also confirmed by our logistic regression model which identifies the motion sickness raw score as a “not successfully adapted” predictor. Interestingly, a recent study conducted on the predictors of motion sickness in parabolic flights has shown that participants who vomited had significantly higher MSSQ scores, but concluded that the MSSQ failed as a vomiting predictor (Golding et al., 2017). In their study, Golding et al. (2017) used the binary variable “vomiting versus no vomiting” in their predictor model. In our study, as well as in a previous study (i.e., Collado et al., 2017), individuals who successfully adapted showed significant differences with individuals who manifested at least one symptom of motion sickness (e.g., sweating, drowsiness, headache, stomach discomfort, nausea, vomiting) and a not exclusively vomiting symptom. Thus, the binary variable “vomiting versus no vomiting” used by Golding et al. (2017) may not be sufficiently discriminating, and this distinction criterion could have been broader (presence or absence of motion sickness symptoms), given that our results supported this. Moreover, on the basis of Golding’s average and conversions table (Golding, 1998), both groups of our subjects appear to be less sensitive than the general population average. This result agrees with previous studies conducted on motion sickness, which have shown low motion sickness susceptibility in participants in parabolic flights compared with the general population, and which have carried out a self-selection of the volunteers (Gaudeau et al., 2002; Golding, 2006; Golding et al., 2017). Nevertheless, although both groups are below Golding’s average, there appear to be two levels of adaptation. Individuals with very low MSSQ-Short scores have adapted successfully to this particular situation, while the environment may have been too novel and disruptive for the others. Thus, it seems that individuals in the lowest 10th percentile of Golding’s conversions table will have no trouble adapting to the demanding situation of parabolic flights.

As far as the Sensation Seeking Scale is concerned, the Adaptive group scored significantly higher in Boredom Susceptibility than the Maladaptive group. As far as the other subscales are concerned, it should be noted that the lack of any difference for Experience Seeking and Thrill and Adventure Seeking does not seem surprising for a population that had been described previously as sensation seekers (Collado et al., 2014; Montag et al., 2016). However, Boredom Susceptibility could be a more subtle behavioral characteristic that would have made individuals in the Adaptive group more dynamic and proactive in their sensation seeking. In fact, Boredom Susceptibility is described as an intolerance to monotony with an aversion to repetitive and routine activities (Zuckerman et al., 1978; Carton et al., 1992). Individuals with a high Boredom Susceptibility score would therefore be regularly looking for new activities. According to Zuckerman (1971), this subscale “incorporates the need for change and variety more than any of the other factors.” In our study, individuals of the Adaptive group may be more accustomed to seeking and experimenting with all sorts of new activities that are related to sensation seeking. This habit, which regularly exposes members of the Adaptive group to new situations, could have led them to develop a certain degree of flexibility when faced with the parabolic flights situation. Given that previous studies from a larger area highlighted the resilient characteristic of sensation seeking behavior (e.g., Engel-Yeger et al., 2016), our results may suggest a ‘protective’ effect on a particular subscale of Sensation Seeking (i.e., the Boredom Susceptibility). Thus, and as corroborated by the logistic regression result, such novelty seeking behavior could, in the context of parabolic flights, have favored the subjects in the Adaptive group to adapt compared to the individuals in the Maladaptive group.

Overall, the individuals who successfully adapted in parabolic flights appear to be more susceptible to boredom with an aversion to routine activities and less susceptible to motion sickness than individuals with difficulties adapting. These dispositional characteristics could have preserved individuals of the Adaptive group faced with the challenging and unusual parabolic flights situation. Although the difference was not significant, coping strategies also seem to distinguish both groups, which have clearly shown differences of symptoms manifested during parabolic flights. As parabolic flights constitute a particular situation in which it is difficult to have a direct action on the “problem” (i.e., different gravity transitions), use of a problem-focused strategy may not have been advantageous in this context. Nevertheless, caution is needed given the small samples of our study but also the use of an abbreviated version of a dispositional coping questionnaire. This result must be completed and refined in future studies with a larger sample and/or another trait coping questionnaire. Moreover, because the quality of adaptation in parabolic flights could be multifactorial, further data are required in order to investigate the involvement of other psychological parameters such as state coping strategies or motivation but also other characteristics such as degree of adaptation (parabolic) flight experience or gender. As part of the development and use of a potential tool to prevent adaptation difficulties in parabolic flights, the results of this exploratory study suggest that it is necessary to consider (1) out of all the motion sickness symptoms (not just vomiting), the presence of at least one characteristic symptom of motion sickness that may reveal the beginnings of difficulties and, (2) the dispositional characteristics of the candidate for parabolic flights. In addition, in future studies, it would be interesting to develop a more suitable tool in order to subtly detect the degree of adaptation difficulties under parabolic flight conditions. A tool like this could refine the detection of adaptation difficulties in parabolic flights and be used for future research. Moreover, in order to achieve a powerful statistical model of predictability, future investigations need to recruit a very large number of subjects. This would lead to substantial, homogeneous groups with different degrees of adaptation difficulties. Nevertheless, because very few studies have been conducted on the psychological aspects of parabonauts, this study enhances the database on the dispositional characteristics of parabonauts and could help to improve the selection of participants for experimental research and/or to adapt the design of future research but could also help prevent the risk of maladaptation and improve the well-being of (future) commercial or occupational aerospace passengers. Finally, the results of this present study primarily confirm the need to consider the quality of adaptation, which is likely to influence the behavior of individuals involved in parabolic flight studies, or more broadly, in extreme environments with high physical demands such as deep-sea diving or very high altitude expeditions.

## Author Contributions

AC, J-PH, VM, and BB: conceived, designed, and performed the research. AC: analyzed the data. AC and BB: contributed to the writing of the manuscript.

## Conflict of Interest Statement

The authors declare that the research was conducted in the absence of any commercial or financial relationships that could be construed as a potential conflict of interest.
